# The interaction effects of automation and population aging on labor market

**DOI:** 10.1371/journal.pone.0263704

**Published:** 2022-02-08

**Authors:** Piyachart Phiromswad, Sabin Srivannaboon, Pattarake Sarajoti

**Affiliations:** 1 Center of Excellence in Management Research for Corporate Governance and Behavioral Finance, Sasin School of Management, Chulalongkorn University, Bangkok, Thailand; 2 Research Unit in Finance for Sustainability in Disruption Era, Sasin School of Management, Chulalongkorn University, Bangkok, Thailand; University of Jyvaskyla, FINLAND

## Abstract

Automation and population aging are two major forces that will shape the nature of works in the future. However, it is not clear how these forces will interact with each other and affect the labor market. This paper examines the interaction effects of computerization and population aging on the labor market. We found that computerization and population aging have large and statistically significant effects on employment growth but not earnings growth. Also, their interaction terms are statistically significant only for employment growth but not for earnings growth.

## Introduction

Over the past decades, the labor market has been undergoing substantial changes in various aspects. Two of the major forces that have been shaping its landscape are namely i) automation and ii) population aging. The rise of automation, which includes, for example, the use of robots in manufacturing, the computerization of routine tasks in normal operations, and the adoption of artificial intelligence are commonly predicted [1–5]. However, the general perception of the public about this issue tends to be somewhat negative. In particular, many people fear that these automation “robots” may reduce the number of “human” jobs that can be routinely computerized and create mass unemployment [1, 6–9]. For example, Frey and Osborne [1] found that 47% of the jobs in the United States could be computerized and be performed by machine. Nevertheless, many researchers believe that the fear of automation is overrated and automation can complement the existing jobs. Some argue that automation can have positive effects on the labor market and the economy [10–13]. For instance, automation can create new jobs that do not exist before the automation adoption as well as increase labor productivity and thus promote economic growth [11].

Population aging is the second inevitable force. According to the UN report on the World Population Prospects 2019, population aging is the process by which older individuals become a proportionally larger share of the total population. This happens because of the low birth rate and longer life expectancy. Many countries such as the U.S. and the U.K. will approach, what is called, “the super-aged society”; a society where more than 20% of their total population is aged 65 years and older, by the years 2025 and 2030 respectively, while some countries such as Japan, Italy, and Germany have been already there [14]. Several studies examined the impact of population aging on various aspects of the economy (e.g., Acemoglu and Restrepo [15]; Cai and Stoyanov [16]). For example, Cai and Stoyanov [16] classified industries into three groups: age-appreciated, age-neutral, and age-depreciated industries and found that population aging can change the comparative advantage of these industries as measured by the import and export data.

It is not clear how these forces will interact with each other and affect the labor market. For example, construction laborer is an occupation that population aging could negatively affect its labor productivity due to skills deterioration (following Cai and Stoyanov [16] construction laborer is an age-depreciated occupation as it requires physical skills which deteriorate with age). However, as many tasks in construction are routine, these could be computerized and have them be done by robots (e.g., the robot “Spot” from Boston Dynamics could be programmed to support many construction tasks). Therefore, automation could help soften the negative impact on labor productivity in this case. On the other hand, consider healthcare workers, firefighters, and policemen in which population aging could negatively affect their labor productivity due to skills deterioration as these jobs also require physical skills which deteriorate with age. Unfortunately, it is difficult to computerize the tasks of these jobs as they are not routine and involve social interactions. In this case, automation could not help soften the impact on labor productivity. Thus, the impact of automation and population aging on the labor market could greatly depend on how these two forces interact with each other.

Only a few studies examined the effect of computerization and population aging on the labor market. For example, Acemoglu and Restrepo [15] examined the relationship between economic growth, population aging, and automation at the country level. However, they did not consider the interaction effects. This paper is the first paper that examines the interaction effects of computerization and population aging on the U.S. labor market. Our measure of computerization is based directly on the data from Frey and Osborne [1] who had constructed the probability of computerization for 702 occupations. To measure the impact of population aging on the labor market, we followed Cai and Stoyanov [16] who classified occupations into three groups: age-appreciating, age-neutral, and age depreciating occupations. They conjectured that occupations that are age-appreciating will benefit (in terms of gaining comparative advantage) from population aging as these occupations use skills that appreciate by age. On the other hand, occupations that are age-depreciating will be negatively affected by population aging as these occupations use skills that depreciate by age. We used the data from the Occupational Information Network or O*NET which describes the characteristics of each occupation to construct indices of age-related abilities. We examined the impact on the labor market in two dimensions: employment growth and earnings growth at the occupation level. Our data is from the U.S. Current Population Survey from the Bureau of Labor Statistics (BLS). In total, we have 501 occupations with complete data.

We found that both computerization and population aging have large and statistically significant effects on employment growth but not earnings growth. We found strong evidence that computerization negatively affects employment growth. We also found strong evidence that the disaggregated measures of age-related abilities affect employment growth but not the aggregate measure. For the interaction effects, we found strong evidence that the interaction terms play an important role in affecting employment growth. This implies that the effects of computerization on employment depend on the level of each disaggregate age-related ability. The impact of computerization on employment for some occupation groups can even be positive when the positive interaction effect outweighs the direct negative effect of computerization. Thus, we have documented an important complementary role of computerization which works for certain age-related abilities, which we further explain in Section IV Results. Section II discusses the literature review. Section III presents our data and the method that we use. Section V shows the conclusion.

## Literature reviews

Several researchers have examined the impact of automation or computerization on the labor market. Frey and Osborne [1] estimated the probability of computerization for 702 occupations as classified by the Standard Occupational Classification (SOC) of the U.S. Department of Labor. Using both a subjective evaluation from machine learning experts and the data from the O*NET, they found that about half (47 percent) of employment in the U.S. is subjected to a high risk of computerization. They defined computerization based on the question “can the tasks of this job be sufficiently specified, conditional on the availability of big data, to be performed by the state-of-the-art computer-controlled equipment” in which they asked the machine learning experts. Broadly speaking, this definition depends on the current understanding of the machine learning experts on what computers can do. Pajarinen et al. [17] argued that this definition encompasses technologies such as “machine learning” (including “data mining, digital sensing and actuation, machine vision, computational statistics and other sub-fields of artificial intelligence”), “mobile robotics”, and “task restructuring”. Pajarinen et al. [17] extended the analysis of Frey and Osborne [1] to Finland and Norway and found that one-third of employment in Finland and Norway is subjected to a high risk of computerization. Brzeski and Burk [18] examined the impact of computerization on Germany’s labor market using Frey and Osborne’s estimated probability data and found that 59 percent is subjected to a high risk of computerization. Fuei [19] also did a similar analysis in Singapore and found that one-fourth of Singaporean employment is subjected to a high risk of computerization.

Other researchers considered alternative approaches in examining the impact of computerization on the labor market. Autor et al. [20] used the Princeton Data Improvement Initiative (PDII) survey which provides data at job activities level rather than occupation level. The data also focuses on the skills (such as cognitive or physical skills) required to complete these activities. They classified tasks (and then aggregate them to occupation level) into abstract, routine, and manual tasks. They found that their task-level approach provided additional explanatory power for earnings beyond the occupation-level approach. Motivated by the inability that existing models are unable to explain several puzzling features in the labor market such as job polarization and technology-induced offshoring, Acemoglu and Autor [21] provided a theoretical task-based model which allows endogenous determination of skills (either man or machines) for tasks as well as endogenous technological development which is influenced by the labor market condition. Arntz et al. [4, 22] used the task-based approach and estimated the risk of automation of occupations for 21 OECD countries. They explored this issue using the data from the Programme for the International Assessment of Adult Competencies (PIAAC) which contains tasks-based level skills data. They found that only 9 percent of occupations are at risk of automation for 21 OECD countries. They argued that the approach of Frey and Osborne [1] overestimates the impact of computerization on the labor market as it does not incorporate the fact that computerization does not completely replace an occupation but rather replaces some specific tasks within each occupation. Brynjolfsson et al. [12] and Brynjolfsson and Mitchell [23] expanded the scope of exploring the impact of computerization on the labor market to include the impact specifically from machine learning technology. They found that machine learning technology affects occupations in different ways compared to automation technology. For most occupations (around 600 out of 964 occupations), machine learning technology can replace no more than 20 percent of tasks in each of these occupations. They also found that the suitability of the machine learning index has an extremely low correlation with wages. Acemoglu and Restrepo [10] and Graetz and Michaels [5] used actual application of robot data from the International Federation of Robotics. An advantage of this approach is that it captures real applications of some automation technologies. However, due to the limitation of this dataset, it covers only automation in the form of industrial robots which is a subset of all automation technologies.

Another important and related trend is the impact of population aging on the labor market. Attanasio, Kitao, and Violante [24] divided countries into the north region (developed countries) and the south region (developing countries) and constructed population projection in these countries. They argued that three major demographic trends will occur in the future which are i) a significant increase in longevity, ii) significant decrease in the population growth, and iii) a significant increase in retired population from the baby-boom generation. The north will have higher life expectancy than the south but the gap will decline sharply during next 100 years. For the population growth, they projected that the north will slightly have higher population growth as the fertility rate is likely to increase somewhat in the future. This is because the fertility rate in developed countries is very low and the government in many countries will initiate several policies to boost the fertility rate. However, the south will experience a decrease in population growth rate and, in some cases, even a decrease in population. Finally, both the north and the south will experience a significant increase in the retired population as well as a shrinking labor force. This could be an important channel that population aging affects the labor market.

Maestas et al. [25] examined the impact of population aging on economic growth using the state’s data from the U.S. They found that population aging could impact economic growth through the labor market channel by affecting i) labor productivity and ii) labor supply growth. Changing demand patterns for goods and services as the population gets older could be another channel that population aging affects the labor market [26]. In this case, goods and services from sectors that have growing demand due to changing population structure (e.g., adult diapers) would experience a positive employment and earnings effect. The opposite would be the case for sectors that have shrinking demand due to changing population structure (e.g., baby diapers). Wongboonsin and Phiromswad [27] examined the channels that demographic structure could affect economic growth. They found that the channels that demographic structure affects economic growth are different between developed countries and developing countries. They found that investment, institutions, and education are key channels for developed countries. For developing countries, they found that investment, financial market, and trade are key channels. Thus, these could be the channels that demographic structure affects the labor market as well.

Cai and Stoyanov [16] examined the impact of population aging on the comparative advantage of countries by examining the import and export pattern. They argued that aging can have both positive and negative effects on different skills and, thus, different tasks for each occupation. Age-appreciated occupations are those that tend to use skills and abilities that improve with age. For example, oral comprehension and oral expression are rated as two of the top age-appreciated skills which tend to be concentrated among sales representatives and writers. On the other hand, age-depreciated occupations are those that tend to use skills and abilities that decrease with age. For example, coordination (defined as adjusting actions in relation to others’ actions.) and divided attention are two of the top age-depreciated skills which tend to be concentrated among machine-operated engineers. With this information, they classified industries into three groups: age-appreciated, age-neutral, and age-depreciated industries and found that population aging affects the comparative advantage of countries. Meissner, Keitel, Sudmeyer, and Pollok [28] found that older employees are better at “motor sequence learning” than middle-aged employees. Thus, this could be another explanation for some jobs and some industries that experience age appreciation.

## Data and method

To examine the interaction effects of computerization and population aging on the labor market, we use the data from the Current Population Survey (CPS) from the U.S. Bureau of Labor Statistics to measure the employment growth and wage growth which are our two dependent variables. Our data starts from 2011 to 2019. We choose to start our data in 2011 as we want to exclude the subprime mortgage crisis period. Also, we choose to end our data in 2019 as we want to exclude the Covid-19 crisis period (which started to impact the U.S. economy in March 2020).

To measure the degree of computerization, we use the probability of computerization from Frey and Osborne [1]. They have constructed the probability of computerization of 702 occupations based on the classification of the U.S. Labor Department’s Standard Occupational Classification (SOC). They combined both subjective and objective rankings to measure the probability of computerization of each occupation. For the subjective ranking approach, they asked machine learning experts in a workshop to assess the degree of computerization of 70 occupations (0 = not computerizable at all and 1 = completely possible to computerized). For the objective ranking approach, they used information from the O*NET which they defined as “bottlenecks to computerization”. Based on the O*NET data which describe the detail of the knowledge, skills, and abilities that are required to perform a certain occupation, they focused on the job descriptions related to i) perception and manipulation (e.g., finger dexterity), ii) creative intelligence (e.g., originality) and iii) social intelligence (e.g., persuasion and negotiation) to capture the bottlenecks to computerization of each occupation. Occupation with a higher degree of bottlenecks to computerization will have a lower probability of computerization holding the subjective measures. To combine the subjective and objective approaches, they used the subjective ranking data from the machine learning experts as training data for probability classification models which compute the probability of computerization of each occupation. Examples of some occupations that have a high probability of computerization are telemarketers, insurance underwriters, and tax preparers (all with a 99 percent probability of computerization). On the other hand, examples of some occupations that have a low probability of computerization are physicians and surgeons (0.42 percent), dentists (0.44 percent), and human resource managers (0.55 percent).

To measure the impact of population aging on the labor market, we followed Cai and Stoyanov [16] and classified occupations into three groups: age-appreciating, age-neutral, and age depreciating occupations. As mentioned above, aging can have both positive and negative effects on different skills (i.e., cognitive or physical skills) and, thus, on employment and earnings growth of different occupations. We used the data from the Occupational Information Network (O*NET) database to construct 4 variables which are age-appreciated cognitive ability, age-depreciated cognitive ability, age-depreciated physical ability, and lastly age-composite ability. These variables aim to measure the importance of various abilities in completing tasks of each occupation and are defined as functions of the scores of the importance of certain abilities. Table 1 summarizes the constructions of these 4 variables.

**Table 1:** Variable constructions.

Variables	(1) Age-appreciated cognitive ability	(2) Age-depreciated cognitive ability	(3) Age-depreciated physical ability	(4) Age-composite ability
**Operations**	Average of	Average of	Average of	Difference between
**Abilities**	Oral comprehension	Memorization	Dynamic flexibility	(1)
	Oral expression	Time-sharing	Dynamic strength	[(2)+(3)]/2
	Written comprehension	Perceptual speed	Explosive strength	
	Written expression	Speed of closure	Extent flexibility	
			Gross body coordination	
			Gross body equilibrium	
			Stamina	
			Static strength	
			Trunk strength	

As can be seen from Table 1, age-appreciated cognitive ability reflects cognitive abilities that tend to improve with age, for example, oral comprehension. These are abilities that we generally perform better as we grow older. On the other hand, age-depreciated cognitive ability and age-depreciated physical ability reflect abilities that are argued to decrease with age. For example, as we are older, our memory and body strength tend to deteriorate. To examine the net effect of the positive and negative abilities, the age-composite ability is defined as the difference between age-appreciated (cognitive) ability and the average of age-depreciated cognitive ability and age-depreciated physical ability.

## Results

This section presents our empirical results. The objective of our analysis is to examine the impact of computerization and population aging on the labor market. We examine the impact on the labor market in two dimensions, namely employment growth and (weekly) earnings growth. In particular, we also give special attention to the interaction effects of computerization and population aging on the labor market.

Table 2 summarizes the key characteristics of the 2-Digit SOC2010 occupation groups in terms of the probability of computerization, age-composite ability, age-appreciated cognitive ability, age-depreciated cognitive ability, age-depreciated physical ability, average employment, average weekly earnings, and share of workers aged 55+ in the total number of employed workers in the year 2019. All 2-Digit SOC2010 statistics are the average of the associated 6-Digit SOC2010. In total, there are 501 occupations at 6-Digit SOC2010 that we used in this paper.

**Table 2:** Summary statistics for the 2-Digit SOC2010 occupation groups.

(i)	(ii)	(iii)	(iv)	(v)	(vi)	(vii)	(viii)	(ix)	(x)
2-Digit SOC 2010	Occupation Groups	Prob. of Com	Age-composite ability	Age-appreciated cognitive ability	Age-depreciated cognitive ability	Age-depreciated physical ability	Employed 2019 (1,000s)	Weekly Earnings In 2019 (USD)	Share of workers 55+ in 2019
11	Management occupations	17.38	49.65	74.82	42.33	8.02	18,953	1,454.86	29.85
13	Business and financial operations occupations	53.46	50.38	72.07	38.75	4.63	7,904	1,257.28	24.11
15	Computer and mathematical occupations	23.07	46.44	67.73	39.47	3.11	5,351	1,857.80	16.33
17	Architecture and engineering occupations	20.41	47.36	71.86	43.33	5.67	2,323	1,472.38	23.85
19	Life, physical, and social science occupations	33.76	48.28	74.17	41.84	9.95	1,482	1,305.38	22.47
21	Community and social service occupations	6.91	49.74	75.83	40.12	12.06	2,648	968.17	26.40
23	Legal occupations	49.93	58.62	79.06	38.71	2.17	1,954	1,481.00	31.68
25	Education, training, and library occupations	28.83	43.50	71.22	41.15	14.28	9,456	1,047.89	23.02
27	Arts, design, entertainment, sports, and media occupations	24.55	42.64	69.74	39.44	14.76	3,233	1,203.09	21.59
29	Healthcare practitioners and technical occupations	17.05	39.23	71.41	44.27	20.09	9,178	1,317.50	22.19
31	Healthcare support occupations	54.18	32.88	64.00	36.33	25.92	3,634	661.00	20.42
33	Protective service occupations	34.06	27.72	65.90	44.31	32.04	3,008	1,083.29	18.18
35	Food preparation and serving related occupations	78.90	20.03	50.98	33.90	28.01	8,370	531.91	12.78
37	Building and grounds cleaning and maintenance occupations	73.61	19.80	52.78	31.65	34.31	5,746	652.67	27.55
39	Personal care and service occupations	46.68	28.61	57.79	34.58	23.78	5,802	618.00	23.94
41	Sales and related occupations	61.53	43.34	66.86	36.29	10.76	14,742	907.17	24.16
43	Office and administrative support occupations	83.65	41.07	64.96	36.48	11.31	16,940	785.50	25.18
45	Farming, fishing, and forestry occupations	78.33	14.50	49.65	36.02	34.29	1,156	561.00	21.89
47	Construction and extraction occupations	72.09	10.02	48.42	36.75	40.06	8,308	893.60	18.95
49	Installation, maintenance, and repair occupations	65.73	16.37	54.00	40.92	34.33	4,862	980.82	22.89
51	Production occupations	81.81	16.88	49.59	35.75	29.68	7,991	752.92	24.58
53	Transportation and material moving occupations	69.58	19.00	54.38	40.48	30.26	9,954	810.47	25.20

This table presents statistics that summarize the key characteristics of the 2-Digit SOC2010 occupation groups in terms of the probability of computerization, age-composite ability, age-appreciated cognitive ability, age-depreciated cognitive ability, age-depreciated physical ability, average employment in 2019 (in thousands), average weekly earnings in 2019, and share of workers aged 55+ in the total number of employed workers in the year 2019. All 2-Digit SOC2010 statistics are the averages of the associated 6-Digit SOC2010. In total, there are 501 occupations at 6-Digit SOC2010 that we used in this paper.

The top three occupation groups with the highest probability of computerization are office and administrative support (83.65%), production (81.81%), and food preparation and serving related (78.90%). On the other hand, the top three occupation groups with the lowest probability of computerization are social service (6.91%), healthcare practitioners and technical (17.05%), and management (17.38%). This is reasonable and consistent with the three bottlenecks to computerization variables proposed by Frey and Osborne [1] namely social intelligence, creativity, and perception, and manipulation. For example, it would be quite difficult to computerize a chief executive officer (CEO) as this job requires a great deal of social intelligence in negotiations with business partners, dealing with internal organizational conflicts in constructive and “human” ways as well as engaging employees to bring out their best. These are something that a computerized process would not be able to mimic (except in the very distant future). On the other hand, the office and administrative support occupation which mostly deals with routine paperwork processes, setting up meetings, taking notes, or delivering documents do not exhibit these bottlenecks to computerization variables. Therefore, it is not difficult to develop a computerized process that replaces (most of) their tasks.

The top three occupation groups with the highest score of the age-composite ability are legal (58.62), business and financial operations (50.38), and community and social service (49.74). These are occupations that are expected to benefit from having an aging workforce. Primarily, these occupations require the use of cognitive skills such as oral comprehension and oral expression which are getting better with age (see Table 2 column iii). Furthermore, these occupations do not need the use of physical skills such as stamina and gross body coordination which deteriorate with age (see Table 2 column v). On the other hand, the top three occupation groups with the lowest score of the age-composite ability are construction and extraction (10.02), farming, fishing, and forestry (14.50), and installation, maintenance, and repair occupations (16.37). These are occupations that are expected to be negatively impacted by having an aging workforce. These occupations require the use of physical skills which deteriorate with age (see Table 2 column v) but do not require cognitive skills such as oral comprehension and oral expression which get better with age (see Table 2 column iii).

The top three occupation groups with the highest share of workers aged 55+ in the total number of employed workers in the year 2019 (i.e., the most aging occupations) are legal (31.68%), management (29.85%), and building and grounds cleaning and maintenance (27.55%). As mentioned above, legal and management are expected to benefit positively from overall skills appreciation from population aging as their scores of the age-composite ability are rather high (58.62 and 49.65 by order). On the other hand, building and grounds cleaning and maintenance is expected to be negatively affected by overall skill deterioration from population aging as their scores of the age-composite ability are rather low (19.80). The three highest-paid (per week) occupations are legal ($1,481), architecture and engineering ($1,472), and management ($1,454). The three largest occupations by the number of workers are management (18.9 million workers), office and administrative support occupations (16.9 million workers), and sales and related occupations (14.7 million workers). These are all consistent with the general structure of the U.S. labor market.

In Table 3, we used linear regression to examine the impact of the probability of computerization and age-related abilities on employment growth. Broadly speaking, we divided the analysis into 2 parts. The first part (columns 1, 3, and 5) uses the aggregate measures of age-related ability (i.e., age-composite ability) in the regressions. The second part (columns 2, 4, and 6) uses the disaggregated measures of age-related ability (namely, age-appreciated cognitive ability, age-depreciated cognitive ability, and age-depreciated physical ability). These analyses will allow us to examine both the aggregate and disaggregate effect of age-related ability on employment growth. Our sample is divided into three periods, full sample period (2011 to 2019 for columns 1 and 2), sub-sample period 1 (2014 to 2019 for columns 3 and 4), and sub-sample period 2 (2011 to 2014 for columns 5 and 6). In addition, we include control variables (which are the share of older workers aged 55+, the share of younger workers aged 16–24, the share of male workers, the share of white workers, and a dummy variable for routine or non-routine occupation) in the estimations of columns 2 and 6, address the issue of outlier using the M‑estimation of Huber [29], and examine the role of the interaction effects. The data for the share of older workers aged 55+, the share of younger workers aged 16–24, the share of male workers, and the share of white workers in the employed population is based on the Current Population Survey (CPS) from the U.S. Bureau of Labor Statistics. For occupations with missing information from the Current Population Survey, we supplement it with the data from the Census Bureau’s American Community Survey (ACS) Public Use Microdata Sample (PUMS) 5-Year estimates. For the dummy variable for routine or non-routine occupation, we follow the classification of Cortes et al. [30] where we grouped their classification into routine and non-routine occupations.

**Table 3:** The impact of the computerization and age-appreciation or age-depreciation on employment.

	Estimates for employment growth from the Census Population Survey					
	2011 to 2019		2014 to 2019		2011 to 2014	
	(i)	(ii)	(iii)	(iv)	(v)	(vi)
Probability of Computerization	-15.45*** (2.51)	-14.34** (6.00)	-11.78*** (4.41)	-8.87* (4.89)	-2.88 (3.60)	-3.44 (4.04)
Age-composite ability	0.05 (0.12)	-	0.02 (0.09)	-	-0.02 (0.08)	-
Age-appreciated cognitive ability	-	0.55** (0.25)	-	0.49** (0.20)	-	-0.19 (0.17)
Age-depreciated cognitive ability	-	-0.10 (0.27)	-	0.007 (0.22)	-	0.21 (0.18)
Age-depreciated physical ability	-	0.43*** (0.15)	-	0.39*** (0.12)	-	-0.12 (0.10)
Prob of Com X Age-composite ability	0.21 (0.75)	-	0.24 (0.23)	-	-0.09 (0.18)	-
Prob of Com X Age-appreciated cognitive ability	-	2.05*** (0.63)	-	1.95*** (0.51)	-	-0.37 (0.42)
Prob of Com X Age-depreciated cognitive ability	-	-0.69 (0.75)	-	-0.66 (0.61)	-	0.49 (0.50)
Prob of Com X Age-depreciated physical ability	-	1.27*** (0.40)	-	1.20*** (0.33)	-	-0.18 (0.26)
Share Old	-3.34 (16.09)	8.03 (16.00)	-8.60 (13.36)	1.36 (13.27)	-1,99 (10.70)	-4.13 (10.76)
Share Young	-14.17 (14.29)	-11.23 (14.27)	-14.56 (12.00)	-10.57 (12.00)	-6.86 (9.50)	-5.12 (9.60)
Share Male	4.34 (5.97)	-2.31 (6.17)	3.71 (4.80)	-1.91 (5.01)	1.49 (3.97)	2.20 (4.15)
Share White	-29.56** (12.30)	-34.64*** (12.61)	-22.59** (9.11)	-25.22*** (9.39)	-12.13 (8.17)	-12.84 (8.48)
Routine	-14.60*** (3.80)	-13.67*** (3.77)	-9.22*** (3.07)	-7.79*** (3.06)	-9.37*** (2.52)	-9.61*** (2.54)
Correct for outliers	Yes	Yes	Yes	Yes	Yes	Yes
R^2^	0.11	0.13	0.07	0.10	0.04	0.05
Multicollinearity test	Pass	Pass	Pass	Pass	Pass	Pass
Number of observations	432	432	434	434	432	432

Notes: This table presents the impact of computerization and age appreciation or age-depreciation skills on employment growth from 2011 to 2019, 2014 to 2019, and 2011 to 2014 from the Census Population Survey. The coefficients with *** are significant at the 1% confidence level; with ** are significant at the 5% confidence level; and with * are significant at the 10% confidence level. For examining the impact of outliers, we use the M‑estimation [29] since, based on visual inspection of the dependent variable, there are several occupations with large negative employment growth. Standard errors are based on Huber’s type I. The age-composite ability variable is the aggregate of age-appreciated cognitive ability and age-depreciated skills (average of cognitive and physical). Explanatory variables in all estimations are entered as deviation from their mean to aid the interpretation of the interaction terms. The multicollinearity test is based on calculating the variance inflation factor (VIF) of each explanatory variable. “Pass” indicates that the VIF is less than 10 which can rule out severe multicollinearity.

For the full sample analysis in Table 3 (columns 1 and 2), we found strong evidence that computerization negatively affects employment growth. In particular, the higher the probability of computerization, the lower the employment growth. We found that the probability of computerization is statistically significant at a 5% confidence level. This is consistent with the previous literature that found a negative effect of automation on employment (e.g., Acemoglu and Restrepo [10]; Graetz and Michaels [5]). For the aggregate measure of age-related ability (age-composite ability), we found no evidence that it affects employment growth (column 1) as the age-composite ability is not statistically significant. However, we found strong evidence that the disaggregated measures of age-related abilities affect employment growth. We found that the disaggregated measures of age-related abilities are statistically significant at least at a 5 percent significance level. In particular, the age-appreciated cognitive ability and age-depreciated physical ability are statistically significant at 5% and 1% level of confidence by order.

An increase in the abilities of workers could have both positive and negative effects on employment. This depends on how fast the marginal productivity of labor rises or falls in relation to the change in the marginal revenue product of labor. As ability increases and shifts production function upward, the labor demand will increase if the marginal revenue product of labor falls slower than the fall in the marginal productivity of labor. On the other hand, as ability increases and shifts production function upward, the labor demand will fall if the marginal revenue product of labor falls faster than the fall in the marginal productivity of labor.

We found that age-appreciated cognitive ability has a positive effect on employment growth. This could imply that, for occupations that depend a lot on an age-appreciated cognitive ability such as legal occupations as well as community and social service occupations, the workers become more productive as they aged and drive up the demand for workers (as marginal revenue product of labor falls slower than the fall in the marginal productivity of labor) holding other factors constant. Furthermore, we found that age-depreciated physical ability has a positive effect on employment growth. This could imply that, for occupations that depend a lot on an age-depreciated physical ability such as construction and extraction occupations as well as installation, maintenance, and repair occupations, the workers become less productive as they age but this increases the demand for workers (as marginal revenue product of labor falls faster than the fall in the marginal productivity of labor) holding other factors constant.

Examining the interaction terms in Table 3 column 2, we found strong evidence that the interaction terms play an important role in affecting employment growth. This is consistent with the indication that both computerization and population aging (via age-related abilities), together, play an important role in shaping the future of the labor market. We found that the interaction effects of i) the probability of computerization and the age-appreciated cognitive ability and ii) the probability of computerization and the age-depreciated physical ability are statistically significant at a 1% level of confidence. On the other hand, the interaction effects of the probability of computerization and age-depreciated cognitive ability is statistically insignificant.

This implies that the effect of computerization on employment depends on the level of age-related abilities. In particular, for occupations with a low score in both age-appreciated cognitive ability as well age-depreciated physical ability (such as production occupations and food preparation and serving related occupations), the negative effect of computerization on employment tends to be strongest. However, for occupations with a high score in both age-appreciated cognitive ability as well as age-depreciated physical ability (such as protective service occupations and healthcare practitioners and technical occupations), the negative effect of computerization on employment tends to be weakest. The impact of computerization on employment for the latter group of occupations can even be positive. Thus, we have documented an important complementary role of computerization which works for certain age-related abilities.

Table 3 columns 3 and 4 examine the impact of the probability of computerization and age-related abilities on employment growth from 2014 to 2019. We found similar evidence for the impact of computerization and age-related abilities on employment growth as reported earlier. In particular, there is strong evidence that computerization negatively affects employment growth as well as there is strong evidence that the disaggregated measures of age-related abilities—not the aggregate measure—affect employment growth. We found that the age-appreciated cognitive ability and age-depreciated physical ability are statistically significant at least a 5% level of confidence. For the interaction terms in Table 3 column 4, we found similar evidence that the interaction terms play an important role in affecting employment growth. We found that the interaction effects of i) the probability of computerization and the age-appreciated cognitive ability and ii) the probability of computerization and the age-depreciated physical ability are statistically significant at a 1% level of confidence. This implies that the effect of computerization on employment depends on the level of age-related abilities as discussed earlier.

Interestingly, in Table 3 columns 5 and 6 which examine the impact of computerization and age-related abilities on employment growth from 2011 to 2014, we do not find any evidence for the impact of the probability of computerization and age-related abilities on employment growth. One explanation for this finding is that the impact of computerization and age-related abilities on employment growth has occurred only recently (i.e., in the past 5 years). This is consistent with the notion that disruptive technologies and changes in age-related abilities only start to have a noticeable impact on the labor market (and possibly, the economy).

Table 4 examines the impact of the probability of computerization and age-related abilities on earnings growth for the full sample period (2011 to 2019 for columns 1 and 2), sub-sample period 1 (2014 to 2019 for columns 3 and 4), and sub-sample period 2 (2011 to 2014 for columns 5 and 6). For the impact of computerization on earnings growth, we do not find any evidence as the estimated coefficients for the probability of computerization are statistically insignificant in all specifications. Similarly, for the impact of age-related abilities on earnings growth, we do find only weak evidence that a disaggregate measure of age-related abilities (namely, the age-depreciated cognitive ability) affects earnings growth negatively (only at a 10% level of confidence). For the interaction effects, there is no evidence to support the importance of the interaction effects of computerization and age-related abilities on earnings growth (except for the 2011 to 2014 period in which we find that interaction effect of the probability of computerization and the age-depreciated physical ability are statistically significant at a 5% level of confidence).

**Table 4:** The impact of the computerization and age-appreciation or age-depreciation on wages (median weekly earnings).

	Estimates for wages growth from the Census Population Survey					
	2011 to 2019		2014 to 2019		2011 to 2014	
	(i)	(ii)	(iii)	(iv)	(v)	(vi)
Probability of Computerization	0.54 (2.45)	-0.49 (2.66)	0.65 (2.31)	0.74 (2.52)	0.52 (1.76)	0.79 (1.92)
Age-composite ability	0.05 (0.05)	-	0.04 (0.05)	-	0.04 (0.04)	-
Age-appreciated cognitive ability	-	-0.02 (0.11)	-	-0.02 (0.10)	-	0.08 (0.08)
Age-depreciated cognitive ability	-	-0.11 (0.12)	-	0.08 (0.11)	-	-0.15* (0.08)
Age-depreciated physical ability	-	-0.09 (0.07)	-	-0.05 (0.06)	-	-0.03 (0.05)
Prob of Com X Age-composite ability	-0.06 (0.13)	-	-0.10 (0.12)	-	-0.000 (0.09)	-
Prob of Com X Age-appreciated cognitive ability	-	-0.47 (0.28)	-	0.06 (0.26)	-	-0.38 (0.20)
Prob of Com X Age-depreciated cognitive ability	-	0.44 (0.35)	-	-0.02 (0.32)	-	0.05 (0.24)
Prob of Com X Age-depreciated physical ability	-	-0.27 (0.19)	-	0.16 (0.18)	-	-0.31** (0.13)
Share Old	-7.54 (9.05)	-11.41 (9.14)	0.16 (8.99)	1.24 (9.08)	-4.37 (6.58)	-5.21 (6.65)
Share Young	22.65*** (7.27)	21.26*** (7.52)	14.70** (7.04)	15.33** (7.20)	10.94** (4.91)	11.07** (5.12)
Share Male	6.00** (2.59)	7.29*** (2.77)	1.59 (2.34)	0.83 (2.54)	4.11** (1.86)	5.95*** (1.98)
Share White	-17.84*** (5.88)	-15.25** (6.47)	-11.67** (5.09)	-12.19** (5.51)	-6.63 (4.28)	-5.81 (4.64)
Routine	-2.07 (1.57)	-2.22 (1.59)	-1.11 (1.46)	-1.23 (1.48)	-1.28 (1.10)	-1.05 (1.11)
Correct for outliers	Yes	Yes	Yes	Yes	Yes	Yes
R^2^	0.11	0.13	0.04	0.05	0.05	0.06
Multicollinearity test	Pass	Pass	Pass	Pass	Pass	Pass
Number of observations	266	266	268	268	270	270

Notes: This table presents the impact of computerization and age-appreciation or age-depreciation skills on wages growth from 2011 to 2019, 2014 to 2019, and 2011 to 2014 from the Census Population Survey. The coefficients with *** are significant at the 1% confidence level; with ** are significant at the 5% confidence level; and with * are significant at the 10% confidence level. For examining the impact of outliers, we use the M‑estimation [29] since, based on visual inspection of the dependent variable, there are several occupations with large negative employment growth. Standard errors are based on Huber’s type I. The age-composite ability variable is the aggregate of age-appreciated cognitive ability and age-depreciated skills (average of cognitive and physical). Explanatory variables in all estimations are entered as deviation from their mean to aid the interpretation of the interaction terms. The multicollinearity test is based on calculating the variance inflation factor (VIF) of each explanatory variable. “Pass” indicates that the VIF is less than 10 which can rule out severe multicollinearity.

One possible explanation for this finding is that computerization and age-related abilities do not have any effect on earnings growth is due to wage rigidity. For example, Haefke et al. [31] used the Current Population Survey (CPS) from the U.S. Bureau of Labor Statistics and found that the wage elasticities of existing workers are relatively small. Several researchers provide various explanations for wage rigidity which include adverse selection problems (e.g., Campbell III et al. [32]) and labor law and collective bargaining power (e.g., Holden [33]; Holden [34]).

Table 5 examines the robustness of the above findings by estimating the impact of the probability of computerization and age-related abilities on employment growth and earnings growth using seemingly unrelated regression (SUR) estimation and three stages least square (3SLS) estimation. A major advantage of the seemingly unrelated regression (SUR) estimation over the OLS estimation is that the SUR estimation takes into account the cross-equation correlation of error terms which tends to make the SUR estimation more efficient than the OLS estimation. Table 5 columns 1 to 6 consider the SUR estimations for employment and wages regressions for the full sample period (2011 to 2019 for columns 1 and 2), sub-sample period 1 (2014 to 2019 for columns 3 and 4), and sub-sample period 2 (2011 to 2014 for columns 5 and 6). Based on the above finding, we consider only estimations using the disaggregated measures of age-related abilities. Overall, the above findings remain intact and robust. Table 5 columns 7 and 8 consider the three stages least (3SLS) square estimation for employment and wages regressions for the sub-sample period 1 (2014 to 2019). The three stages least square (3SLS) estimation also retains the advantage that it tends to be more efficient than OLS estimation because it takes into account the cross-equation correlation of error terms. However, another major advantage of the three stages least square (3SLS) estimation is its ability to address the endogeneity problem. We use the previous period employment and earnings growth (2011 to 2014 period) as the instrumental variables for estimating the 3SLS estimation for sub-sample period 1 (2014 to 2019). Overall, most of the above findings remain intact and robust. We still find evidence that the interaction terms play an important role in affecting employment growth. For the earnings growth, we find no evidence that computerization and age-related abilities affect earnings growth. One exception is that the effect of computerization on employment is statistically insignificant (but the sign remains negative).

**Table 5:** Robustness analyses on the impact of the computerization and age-appreciation or age-depreciation on employment and wages (median weekly earnings) based on seemingly unrelated regression (SUR) and three stages least square (3SLS).

	Estimates for employment growth and wages growth (median weekly earnings) from the Census Population Survey							
	SUR 2011 to 2019		SUR 2014 to 2019		SUR 2011 to 2014		3SLS 2014 to 2019	
	Employment (i)	Wages (ii)	Employment (iii)	Wages (iv)	Employment (v)	Wages (vi)	Employment (vii)	Wages (viii)
Probability of Computerization	-15.28** (7.39)	-0.80 (2.72)	-11.27** (5.64)	-3.50 (2.66)	-1.22 (6.13)	2.87 (2.14)	-5.41 (5.32)	-2.89 (2.65)
Age-appreciated cognitive ability	0.57* (0.31)	-0.06 (0.11)	0.52** (0.24)	-0.07 (0.11)	0.14 (0.26)	0.03 (0.09)	0.31 (0.22)	-0.04 (0.11)
Age-depreciated cognitive ability	-0.21 (0.33)	-0.09 (0.13)	-0.25 (0.25)	-0.03 (0.12)	0.08 (0.27)	-0.11 (0.09)	0.20 (0.24)	-0.04 (0.12)
Age-depreciated physical ability	0.23 (0.19)	-0.09 (0.07)	0.47*** (0.14)	-0.07 (0.07)	-0.11 (0.15)	-0.05 (0.05)	0.41*** (0.14)	-0.07 (0.07)
Prob of Com X Age-appreciated cognitive ability	2.00** (0.77)	-0.51* (0.28)	2.10*** (0.59)	-0.09 (0.27)	-0.22 (0.64)	-0.33 (0.22)	1.95*** (0.55)	-0.06 (0.27)
Prob of Com X Age-depreciated cognitive ability	0.10 (0.93)	0.26 (0.36)	-0.95 (0.71)	0.23 (0.34)	1.07 (0.77)	-0.16 (0.26)	-0.17 (0.66)	0.20 (0.35)
Prob of Com X Age-depreciated physical ability	1.19** (0.49)	-0.28 (0.19)	1.31*** (0.38)	0.02 (0.19)	0.09 (0.40)	-0.38** (0.15)	1.53*** (0.38)	0.05 (0.19)
Share Old	-11.07 (19.69)	-15.50* (9.31)	-6.36 (15.33)	-16.67* (9.56)	-13.78 (16.34)	-4.75 (7.42)	12.75 (19.29)	-21.78** (9.77)
Share Young	-36.69** (17.56)	20.47*** (7.66)	-11.45 (13.86)	10.38 (7.58)	-9.97 (14.57)	6.18 (5.72)	-16.28 (14.71)	9.64 (7.75)
Share Male	2.95 (7.60)	6.62** (2.83)	-3.47 (5.78)	0.90 (2.67)	5.51 (6.30)	4.75** (2.21)	-2.60 (5.45)	0.61 (2.71)
Share White	-52.79*** (15.53)	-15.70** (6.60)	-18.71* (10.84)	-6.84 (5.80)	-19.24 (12.88)	-7.95 (5.18)	-12.72 (12.23)	-6.78 (6.12)
Routine	-16.04*** (4.65)	-2.01 (1.62)	-4.68 (3.53)	-0.40 (1.56)	-8.12** (3.85)	-2.16* (1.24)	-7.68** (3.06)	0.05 (1.56)
R^2^	0.16	0.16	0.09	0.07	0.04	0.08	0.15	0.07
Multicollinearity test	Pass	Pass	Pass	Pass	Pass	Pass	Pass	Pass
Number of observations	432	266	434	268	432	270	270	261

Notes: This table presents the impact of computerization and age-appreciation or age-depreciation skills on employment growth and wages growth from 2011 to 2019, 2014 to 2019, and 2011 to 2014 from the Census Population Survey. The coefficients with *** are significant at the 1% confidence level; with ** are significant at the 5% confidence level; and with * are significant at the 10% confidence level. Explanatory variables in all estimations are entered as deviation from their mean to aid the interpretation of the interaction terms. The multicollinearity test is based on calculating the variance inflation factor (VIF) of each explanatory variable. “Pass” indicates that the VIF is less than 10 which can rule out severe multicollinearity.

## Conclusion

Two of the major forces that have been playing an important role in shaping the labor market in the future are automation or computerization and population aging. Several research works have examined the impact of computerization and population aging on the labor market. However, there are no studies that examine and estimate the interaction effects of computerization and population aging on the labor market. This paper is the first paper in this aspect.

Why do the interaction effects matter? It is plausible that automation or computerization could complement or substitute some skills or abilities. For example, construction workers whose skills and abilities deteriorate with age could be replaced by a computerized robot that can perform most of the construction worker tasks better. However, healthcare workers, firefighters, and policemen whose skills and abilities deteriorate with age could benefit from computerization as a computerized robot or process could be used to enhance their productivity rather than replace them. Thus, the effect of computerization or population aging (via age-related abilities) on the labor market could depend on how these two forces interact.

We examine the interaction effects of computerization and population aging (via age-related abilities) on the U.S. labor market. We use the probability of computerization from Frey and Osborne [1] to measure computerization. We follow Cai and Stoyanov [16] and constructed age-related abilities from the Occupational Information Network or O*NET. We consider both the aggregate measure of age-related ability and disaggregate measures of age-related abilities which are age-appreciated cognitive ability, age-depreciated cognitive ability, and age-depreciated physical ability. We examine the impact of computerization and population aging (via age-related abilities) on the labor market in two dimensions: employment growth and (weekly) earnings growth. The data for employment growth and (weekly) earnings growth is from the U.S. Current Population Survey from the Bureau of Labor Statistics (BLS). In total, we have 501 occupations with complete data.

We found that both computerization and population aging have large and statistically significant effects on employment growth but not earnings growth. Specifically, for employment growth, the interaction terms are statistically significant and will play an important role in shaping their impact on the labor market. We found strong evidence that computerization negatively affects employment growth holding other factors constant. This is consistent with the previous literature (e.g., Acemoglu and Restrepo [10]; Graetz and Michaels [5]). We also found strong evidence that the disaggregated measures of age-related abilities affect employment growth but not the aggregate measure.

For the interaction effects, we found strong evidence that the interaction terms play an important role in affecting employment growth. This implies that the effect of computerization on employment depends on the level of each disaggregated age-related ability. In particular, we found that for occupations with a low score in both the age-appreciated cognitive ability as well age-depreciated physical ability (such as production occupations and food preparation and serving related occupations), the negative effect of computerization on employment tends to be strongest. However, for occupations with a high score in both age-appreciated cognitive ability as well as age-depreciated physical ability (such as protective service occupations and healthcare practitioners and technical occupations), the negative effect of computerization on employment tends to be weakest. The impact of computerization on employment for the latter group of occupations through the positive interaction effects is even stronger than the direct negative effect itself which is possible to make the overall impact of computerization on employment for these occupations be positive. Thus, we have documented an important complementary role of computerization which works for certain age-related abilities. This will be important information that can help firms properly adapt to future business conditions, which are technology disruption [1, 11, 12, 35], demographic disruption [15, 16, 24–27, 36], environmental disruption [37, 38], and Covid-19 disruption [39, 40].

## Supporting information

S1 File (DOCX)
