# Supplementary material for: The interaction effects of automation and population aging on labor market
Source: PLoS One. 2022 Feb 8;17(2):e0263704. doi: 10.1371/journal.pone.0263704 (PMC8824351; doi:10.1371/journal.pone.0263704)
Supplement: S1 File — (DOCX) [file pone.0263704.s001.docx]

Data Availability Statement:

There are 3 main datasets for this paper. We could not upload the data because the data are owned by a third-party organization. But it is possible to purchase and/ or obtain the data to replicate the results of this study using the steps below.

• The first one is the probability of computerization from Frey and Osborne (2017). The data can be obtained directly from the following link (see Appendix A):

- <https://doi.org/10.1016/j.techfore.2016.08.019> or
- <https://www.robots.ox.ac.uk/~mosb/public/pdf/5383/Frey_Osborne_2017_The%20future%20of%20employment.pdf>

• The second one is age-appreciated cognitive ability, age-depreciated cognitive ability, age-depreciated physical ability from the Occupational Information Network (O*NET) database. The data can be obtained directly from the following link:

- Age-appreciated cognitive ability is the average of oral comprehension, oral expression, written comprehension and written expression (classified by O*NET as cognitive abilities):

| Variables | Links |
| --- | --- |
| Oral comprehension | <https://www.onetonline.org/find/descriptor/result/1.A.1.a.1?a=1> |
| Oral expression | <https://www.onetonline.org/find/descriptor/result/1.A.1.a.3?a=1> |
| Written comprehension | <https://www.onetonline.org/find/descriptor/result/1.A.1.a.2?a=1> |
| Written expression | <https://www.onetonline.org/find/descriptor/result/1.A.1.a.4?a=1> |

- Age-depreciated cognitive ability is the average of memorization, time sharing, perceptual speed and speed of closure (classified by O*NET as cognitive abilities):

| Variables | Links |
| --- | --- |
| Memorization | <https://www.onetonline.org/find/descriptor/result/1.A.1.d.1?a=1> |
| Time sharing | <https://www.onetonline.org/find/descriptor/result/1.A.1.g.2?a=1> |
| Perceptual speed | <https://www.onetonline.org/find/descriptor/result/1.A.1.e.3?a=1> |
| Speed of closure | <https://www.onetonline.org/find/descriptor/result/1.A.1.e.1?a=1> |

- Age-depreciated physical ability is the average of dynamic flexibility, dynamic strength, explosive strength, extent flexibility, gross body coordination, gross body equilibrium, stamina, static strength, trunk strength (classified by O*NET as physical abilities):

| Variables | Links |
| --- | --- |
| Dynamic flexibility | <https://www.onetonline.org/find/descriptor/result/1.A.3.c.2?a=1> |
| Dynamic strength | <https://www.onetonline.org/find/descriptor/result/1.A.3.a.3?a=1> |
| Explosive strength | <https://www.onetonline.org/find/descriptor/result/1.A.3.a.2?a=1> |
| Extent flexibility | <https://www.onetonline.org/find/descriptor/result/1.A.3.c.1?a=1> |
| Gross body coordination | <https://www.onetonline.org/find/descriptor/result/1.A.3.c.3?a=1> |
| Gross body equilibrium | <https://www.onetonline.org/find/descriptor/result/1.A.3.c.4?a=1> |
| Stamina | <https://www.onetonline.org/find/descriptor/result/1.A.3.b.1?a=1> |
| Static strength | <https://www.onetonline.org/find/descriptor/result/1.A.3.a.1?a=1> |
| Trunk strength | <https://www.onetonline.org/find/descriptor/result/1.A.3.a.4?a=1> |

(Note that age-composite ability is constructed from age-appreciated cognitive ability, age-depreciated cognitive ability and age-depreciated physical ability as described in Table 1)

• The third one is the employment growth, wage growth, share of workers 55+, share of workers 16-24, share of male workers and share of white workers from the Current Population Survey (CPS) from the U.S. Bureau of Labor Statistics. The data can be obtained directly from the following link:

| Variables | Links |
| --- | --- |
| Employment, share of workers 55+, share of workers 16-24 in 2011 | <https://www.bls.gov/cps/aa2011/cpsaat11b.htm> |
| Employment and share of workers 55+, share of workers 16-24 in 2014 | <https://www.bls.gov/cps/aa2014/cpsaat11b.htm> |
| Employment and share of workers 55+, share of workers 16-24 in 2019 | <https://www.bls.gov/cps/aa2019/cpsaat11b.htm> |
| Share of male workers and share of white workers in 2011 | <https://www.bls.gov/cps/aa2011/cpsaat11.htm> |
| Share of male workers and share of white workers in 2014 | <https://www.bls.gov/cps/aa2014/cpsaat11.htm> |
| Share of male workers and share of white workers in 2019 | <https://www.bls.gov/cps/aa2019/cpsaat11.htm> |
| Wage (median weekly earnings) in 2011 | <https://www.bls.gov/cps/aa2011/cpsaat39.htm> |
| Wage (median weekly earnings) in 2014 | <https://www.bls.gov/cps/aa2014/cpsaat39.htm> |
| Wage (median weekly earnings) in 2019 | <https://www.bls.gov/cps/aa2019/cpsaat39.htm> |

For occupations with missing information from the Current Population Survey, we supplement it with the data from the Census Bureau’s American Community Survey (ACS) Public Use Microdata Sample (PUMS) 5-Year estimates. The data can be obtained directly from the following link:

- <https://data.census.gov/mdat/#/>

For the dummy variable for routine or non-routine occupation, we follow the classification of Cortes et al. (2020) where we grouped their classification into routine and non-routine occupations.

**References**

Cortes GM, Oliveira A, Salomons A. Do technological advances reduce the gender wage gap?. Oxford Review of Economic Policy. 2020;36(4):903-24.

Frey CB, Osborne MA. The future of employment: How susceptible are jobs to computerisation? Technological Forecasting & Social Change. 2017; 114:254-80.

National Center for O*NET Development. O*NET OnLine Help: Data Collection Information. O*NET OnLine. Retrieved May 29, 2021, from <https://www.onetonline.org/help/online/data>

U.S. Bureau of labor Statistics Division of Labor Force Statistics. Labor Force Statistics from the Current Population Survey. Retrieved May 29, 2021, from <https://www.bls.gov/cps/>
